# Delays in Initiating Anti-Cancer Therapy for Early-Stage Breast Cancer—How Slow Can We Go?

**DOI:** 10.3390/jcm12134502

**Published:** 2023-07-05

**Authors:** Hikmat Abdel-Razeq, Asem Mansour, Sarah Edaily, Abdulmajeed Dayyat

**Affiliations:** 1 Department of Internal Medicine, King Hussein Cancer Center, Amman 11941, Jordan; so.10227@khcc.jo; 2 Department of Internal Medicine, School of Medicine, The University of Jordan, Amman 11942, Jordan; 3 Department of Radiology, King Hussein Cancer Center, Amman 11941, Jordan; amansour@khcc.jo; 4 Department of Radiation Oncology, King Hussein Cancer Center, Amman 11941, Jordan; abdulmajeeddayyat@gmail.com; 5 Department of Radiation Oncology, Dalhousie University, Halifax, NS B3H 4R2, Canada

**Keywords:** treatment delays, COVID-19, refugees, pandemic, breast cancer, time to treatment

## Abstract

Breast cancer is the most commonly diagnosed cancer among women worldwide, and is a leading cause of cancer-related deaths. When diagnosed at an early stage, appropriate and timely treatment results in a high cure rate and better quality of life. Delays in initiating anti-cancer therapy, including surgical resection, adjuvant/neoadjuvant chemotherapy and radiation therapy are commonly encountered, even in developed health care systems. Existing comorbidities that mandate referral to other services, genetic counseling and testing that may dictate the extent and type of anti-cancer therapy and insurance coverage, are among the most commonly cited factors. However, delays can be unavoidable; for over three years, health care systems across the globe were busy dealing with the unprecedented COVID-19 pandemic. War across hot zones around the globe resulted in millions of refugees; most of them have no access to cancer care, and when/where available, there may be significant delays. Thus, cancer patients across the globe will probably continue to suffer from significant delays in diagnosis and appropriate treatment. Many retrospective reports showed significant negative impacts on different aspects of treatment outcomes and on patients’ psychosocial wellbeing and productivity. In this paper, we review the available data on the impact of delays in initiating appropriate treatment on the outcomes of patients with early-stage breast cancer.

## 1. Introduction

Breast cancer is the most common cancer diagnosed among women worldwide, and is a leading cause of cancer-related deaths. There was an estimated 2.26 million new breast cancer cases and 685,000 deaths reported worldwide in 2020 [1,2,3].

Health care systems across the globe were busy dealing with the unprecedented COVID-19 pandemic [4]. More recently, the Russian invasion of Ukraine is a humanitarian disaster, with a wide-ranging impact on the health of those involved. Not too long ago, the Syrian crisis resulted in millions of refugees; many are suffering in their host countries. Such crises and conflicts have directed the interest away from high-yield early detection and cancer prevention programs like screening mammography. It also resulted in significant delays in the appropriate diagnosis of cancer at its earlier stages, and amplified existing issues and problems such countries already have.

These events, along with the witnessed delays observed in treating chronic illnesses including cancer, have raised concerns about the potential negative impacts these delays may have on the treatment outcomes of cancer patients.

Early detection and breast cancer screening programs have significantly contributed to the better outcomes observed in Western societies. Though less than 5% of breast cancer cases are diagnosed at stage IV of the disease in developed countries [5,6], such rates may exceed 20% in some unprivileged societies [7]. However, even the most developed countries had experienced significant delays in breast cancer diagnoses during the pandemic, or in countries with political conflicts and instabilities [8,9]. Cancer screening programs were paused almost worldwide, causing screening disruptions for a significant period of time [10]. Delays also affected elective cancer therapies including surgery, chemotherapy and radiation therapy [11,12].

In this paper, we review the potential impact that delays have in breast cancer treatment across the continuum, and what these delays may mean to outcomes.

## 2. Materials and Methods

A literature review was performed on previously published research on the impact of delays in anti-cancer treatment on breast cancer outcomes. The search was carried out using PubMed on all original research, meta-analyses and review papers between 1990 and 2022. Additionally, we reviewed related data presented in the last 5 years at major international cancer conferences, like the American Society of Clinical Oncology (ASCO), the European Society of Medical Oncology (ESMO), and the San Antonio Breast Cancer Symposium (SABCS).

## 3. Delays in Breast Cancer Treatment

Delays in cancer treatment have adverse consequences on disease control and mortality. A systematic review and meta-analysis of seven cancers (bladder, breast, colon, rectum, lung, cervix and head and neck), representing 44% of all incident cancers globally, showed a 6–8% increase in cancer-related deaths for each 4-week delay in surgery. This impact is even more marked for some radiotherapies and systemic therapies, with a 9% and 13% increased risk of death for definitive head and neck radiotherapy and adjuvant systemic treatment for colorectal cancer, respectively [13].

The time interval to treatment is a key performance indicator (KPI) utilized by many health care systems and services, and a frequently asked question by patients and their relatives [13,14]. However, definitive and convincing answers based on data are lacking. Though clinical practice guidelines do not present specific time limits, some health care facilities established a time limit from the first evaluation to definite treatment as a strictly followed KPI. However, many factors may still contribute to occasional delays in breast cancer treatment, despite all the efforts [15]. Insurance coverage and existing comorbidities that mandate referral to other clinical services are commonly encountered issues, and may lead to significant delays in initiating treatment. Germline genetic testing and counseling, which may dictate the type of surgery, radiotherapy and even chemotherapy, have become a standard of care recommended by many international guidelines, and involve almost two thirds of newly-diagnosed patients with breast cancer [16,17,18]. Given its availability, germline genetic testing may take days or even weeks before results are available for the decision-making process. However, the recent COVID-19 pandemic was by far the most significant contributing factor that led to significant delays in starting or continuing an already-started treatment plan [19].

The time to treatment (TTT) can be addressed in multiple intervals or phases; the time between diagnosis to upfront surgery (TTS-u), the time between neoadjuvant chemotherapy and surgery (TTS-n), and the time to adjuvant chemotherapy (TTC) or radiotherapy (TTR) after surgery.

## 4. Time to “Upfront” Surgery (TTS-u)

More than 100 years ago, William Halsted stated that “in the early stage of breast cancer, we no longer need the proof that the slightest delay is dangerous” [20]. Though delays in breast cancer treatment have been feared since then, clear answers are lacking. The time to surgery (TTS) and its relation to treatment outcomes has been extensively studied [21,22].

A recent report utilized two independent population-based studies on patients diagnosed with early-stage invasive breast cancer from prospectively collected Surveillance Epidemiology and End Results (SEER) data and National Cancer Database (NCDB) data [21]. Each study assessed the overall survival (OS) as a function of time between diagnosis and surgery at different intervals, after adjusting for patient demographics and tumor-related factors. The intervals tested were ≤30, 31–60, 61–90, 91–120 and 121–180 days in length. Additionally, the disease-specific survival was tested at 60-day intervals. More than 94,000 patients were included; all were older than 65 years, and diagnosed between 1992 and 2009. With each interval delay, the OS was lower (hazard ratio [HR] 1.09, *p* < 0.001). Additionally, breast cancer-specific mortality increased with each 60-day interval (sub-hazard ratio [sHR] 1.26, *p* = 0.03). The NCDB reported on more than 115,000 patients ≥ 18 years old, diagnosed between 2003 and 2005. In this study, the overall mortality HR was 1.10 (*p* < 0.001) for each increasing interval, again with adjustments for demographic-, tumor- and treatment-related factors [21]. More recently, and using a bigger group of patients from the NCDB, Wiener and colleagues identified 373,334 women with stage I-III invasive ductal or lobular breast cancer treated between 2010 and 2014, and followed up through 2019. Surgery was the upfront treatment modality among all of the patients. Patients who received neoadjuvant therapy were not included. The median time to surgery was 30 days, and only 12% of the patients underwent surgery after 8 weeks of diagnosis. An age younger than 45, having Medicaid or no insurance, and lower household income were linked to surgical delays. The 5-year OS for the whole group was high, at 90%. From a multivariable analysis, there was no statistically significant association between the time to surgery and overall survival when surgery was performed between 0 and 8 weeks. However, the small fraction of patients who had surgery beyond the 8-week limit had a significantly worse OS at 5 years compared with those who had the surgery performed between 0 to 4 weeks (HR, 1.15; *p* < 0.001) [23].

## 5. Time to Surgery following Neoadjuvant Therapy (TTS-n)

Neoadjuvant chemotherapy (NAC) is increasingly used to treat patients with breast cancer, especially those with node-positive disease, large tumors and tumors with human epidermal growth factor receptor-2 (HER-2) positivity, and/or hormone receptor negativity [24,25]. Data on the optimal timing of surgery and its impact on treatment outcomes following NAC is conflicting.

A sensitivity analysis of data on 1101 breast cancer patients treated with NAC suggested a worse OS in patients who had surgery more than 8 weeks following the last NAC cycle. In this setting, a time to surgery (TTS-n) of less than 8 weeks had equivalent OS, recurrence-free survival (RFS) and locoregional recurrence-free survival [22]. In a similar study from our institution, a retrospective cohort of 468 breast cancer patients who received NAC with anthracyclines and taxanes was divided into three groups based on the interval between the end of NAC and surgery; <4, 4–8 and >8 weeks. After a median follow up of 3.8 years, all of the groups had an equivalent DFS. However, OS was worse for the group of patients who had their surgeries beyond 8 weeks compared to others [26]. Another study included 463 patients with breast cancer who received NAC between 2011 and 2017, and examined the relationship between the TTS-n and residual cancer burden (RCB) and oncologic outcomes. The median TTS-n was 29 days (range 11–153). From the multivariate analysis, a TTS-n > 6 weeks was independently associated with a worse RFS (HR 3.45; *p* < 0.001) and disease-specific survival (DSS) (HR 2.82; *p* < 0.05). Additionally, a TTS-n > 6 weeks was independently associated with a higher RCB score (*p* < 0.0001) [27]. Table 1 summarizes the results of several other studies that addressed the impact of time to surgery (whether upfront or following neoadjuvant chemotherapy) on treatment outcomes.

## 6. Time to Adjuvant Chemotherapy (TTC)

There is convincing evidence that delayed adjuvant chemotherapy will have a negative impact on outcomes [33,34]. Indeed, delays have a more pronounced negative impact in the more aggressive subtypes of breast cancers, like those with triple-negative (TN)-disease.

In one study from the MD Anderson Cancer Center, 6827 patients with stage I-III breast cancer were categorized into three groups according to time to adjuvant chemotherapy (TTC): ≤30, 31–60 and >60 days; survival outcomes were compared accordingly. Initiation of chemotherapy >60 days after surgery was associated with worse OS (HR, 1.76; 95% CI, 1.26 to 2.46) and RFS (HR, 1.34; 95% CI, 1.01 to 1.76). Patients with HER2-positive and those with TN-disease with TTC > 60 days had the worst survival (HR, 3.09; 95% CI, 1.49 to 6.39 and HR, 1.54; 95% CI, 1.09 to 2.18, respectively), compared to those who initiated treatment within the first 30 days postoperatively [31]. In another study, researchers retrospectively analyzed the data of 687 breast cancer patients with stage I-III TN-disease who had surgery followed by adjuvant anthracyclines or anthracyclines plus taxane-based chemotherapy [35]. The study confirmed worse outcomes for those with delayed chemotherapy; 10-year DFS rates were 81.4%, 68.6%, 70.8% and 68.1% among patients who received chemotherapy at ≤30, 31–60, 61–90 and ≥91 days, respectively (*p* = 0.005). Additionally, the 10-year OS rate decreased with delayed adjuvant chemotherapy: 82%, 67.4%, 67.1% and 65.1% among patients who received chemotherapy at ≤30, 31–60, 61–90 and ≥ 91 days, respectively (*p* = 0.003) [35].

A meta-analysis of eight high-validity studies on breast cancer demonstrated that a 4-week increase in the time to initiate chemotherapy following surgery was associated with a significant increase in the risk of death by 4–8% [36]. A recent study used the National Cancer Database to identify 172,043 patients with stage I-III breast cancer treated with surgery followed by adjuvant chemotherapy between 2010 and 2014. The time interval from diagnosis to surgery and then to adjuvant chemotherapy was studied in relation to survival outcomes. Chemotherapy that was initiated more than 120 days after breast cancer diagnosis was considered a delay. The median time from diagnosis to surgery was 27 days, while the median time between surgery and the initiation of chemotherapy was 43 days for the whole study group. The median time from diagnosis to the initiation of chemotherapy was 74 days, and most patients (89.5%) initiated chemotherapy within the 120-day cutoff. The analysis showed that regardless of the surgery type, patients who started chemotherapy beyond 120 days from the time of diagnosis had worse overall survival compared to patients who started chemotherapy within 120 days (HR = 1.29; 95% CI, 1.22–1.37; *p* < 0.001). This poor outcome was seen across all tumor subtypes, with HER2-positive disease being most affected (HR = 1.47; 95% CI, 1.29–1.68), followed by TN-disease (HR = 1.23; 95% CI, 1.10–1.38) [32]. A more recent study from the Netherlands reached similar conclusions [35], while another study from the University of Pennsylvania had contradicting results [36]. The latter study included 724 early-stage TN breast cancer patients who received adjuvant chemotherapy from 2009 to 2018. The median time from surgery to chemotherapy was 42 days. In a multivariate analysis, a TTC >56 days (*n* = 173) had no negative impact on DFS or OS compared to a TTC ≤31 (*n* = 198) days (*p* = 0.27 and *p* = 0.21, respectively).

Given the above data, the evidence is convincing that delaying adjuvant chemotherapy beyond the first 30 or 60 days is associated with a worse disease recurrence rate and overall survival outcomes. Table 2 summarizes some of the key studies that addressed issues related to the time to chemotherapy.

## 7. Time to Radiotherapy (TTR)

There are conflicting data regarding the optimal interval between surgery and adjuvant radiotherapy (TTR) [40,41,42], as shown in Table 3. In one population-based retrospective cohort study that included a random sample of all breast cancer patients at stage I or II treated over one year (2001–2002) in Ontario, researchers looked at the impact of delays in delivering radiation therapy on composite survival outcomes that included EFS (including locoregional recurrence and distal metastasis) and breast cancer related-mortality. A total of 1028 patients treated with breast-conserving surgery and adjuvant radiation therapy were identified. The patients were analyzed in two groups. In the first group (*n* = 599), patients were treated with adjuvant radiation with no intervening chemotherapy. After a median follow-up of 7.2 years, a TTR of 12 weeks or more was associated with worse EFS (HR for the composite outcome: 1.44; 95% CI: 0.98 to 2.11; *p* = 0.07). In the second group (*n* = 429), patients received intervening adjuvant chemotherapy. A waiting time of 6 weeks or more from completion of chemotherapy to initiation of radiation therapy was associated with worse EFS (HR: 1.50; 95% CI: 1.00 to 2.22; *p* = 0.047) [43].

In another recent retrospective study that reviewed the data of 989 patients, a TTR beyond 12 weeks after chemotherapy was associated with significantly worse breast cancer-specific survival (BCSS) and OS compared to a TTR < 4, 4–8 or 8–12 weeks [44]. A meta-analysis conducted to determine the effect of delays in postoperative radiotherapy on local recurrence found a relative risk of 1.08 (95% CI: 1.02–1.14) for local recurrence per month of delay, without a statistically significant effect on overall survival [45]. These conclusions are similar to findings of an earlier study conducted on a sample of 926 randomly selected women diagnosed with localized breast cancer treated in five regions of Québec, Canada, between 1988 and 1994. The study found that although a longer waiting time to radiotherapy may compromise local control, it did not influence survival at 7 years when other predictors of outcomes are taken into account [46].

For ethical reasons, conducting randomized controlled trials to assess the effect of the time interval between surgery and radiotherapy on survival outcomes is not possible. Therefore, efforts should be exercised to shorten waiting times to avoid potential detrimental consequences, and to minimize patients’ physical and emotional suffering at least for local recurrences.

## 8. Looking Ahead and Conclusions

In this review, we analyzed the potential negative impacts of delaying treatment initiation for patients with breast cancer. Such negative impacts may be encountered at all treatment segments, including upfront surgery, neoadjuvant or adjuvant chemotherapy and adjuvant radiation therapy.

Most studies, however, do not measure the negative impact on other important outcomes, such as functional outcomes, complications and the cost of more extensive treatments that may be needed for disease progression during delays. A population-based cost analysis from Europe even showed a more significant economic burden because of higher direct care costs and productivity losses from premature mortality and morbidity [47]. Therefore, the impacts of treatment delays are probably far more significant for patients and society than those reflected in the disease control and mortality figures. It is anticipated that delays in cancer diagnosis and treatment have a tremendous psychosocial impact; however, much of the literature on treatment delays focused on the physical impacts on patients [47].

## Figures and Tables

**Table 1:** Time to surgery (TTS).

Author/Year [References]	Country	Study Design	Study Period	No. of Patients	Median/ Mean Age	End Point(s)	Findings
Time to Upfront Surgery (TTS-u)							
Vanni G 2020 [9]	Italy	Retrospective, COVID cohort vs. historical pre-COVID cohort	2019–2020	432	62	Breast cancer presentation	Increase in lymph node involvement in the COVID cohort
Bleicher RJ 2012 [15]	USA	Retrospective analysis of prospectively collected SEER data	1992–2005	72,586	75	Factors associated with delays between symptoms and surgery	Certain demographics and preoperative evaluation lead to greater TTS
Bleicher RJ 2016 [21]	USA	Two independent population-based analysis of prospectively collected SEER and NCDB data	1992–2009 (SEER) 2003–2005 (NCDB)	94,544 (SEER) 115,790 (NCDB)	75.2 (SEER) 60.3 (NCDB)	Effect of TTS on OS and DSS	Longer TTS is associated with lower OS and DSS
Eaglehouse YL 2019 [28]	USA	Cross-sectional retrospective study using the U.S. Military Health System database	1998–2010	9669	54.5	All cause death	Longer TTS (≥36 days) associated with poorer OS
Polverini AC 2016 [29]	USA	Retrospective analysis of prospectively collected NCDB data	2004–2012	420,792	59.4	Effect of TTS on OS	Longer TTS (≥8 weeks for stage I, >12 weeks for stage II) associated with decreased OS compared to TTS < 4 weeks
Mateo AM 2020 [30]	USA	Retrospective analysis of prospectively collected NCDB data	2010–2014	351,087	NA	OS for triple negative and other phenotypes	OS decreased for each month of delay by HR 1.104 for all phenotypes
Shin DW 2013 [31]	Republic of Korea	Analysis of prospectively collected data	2006–2011	7529	49.3 (for breast patients)	Effect of TTS on OS	TTS >12 weeks is associated with Increased mortality
Weiner AA 2023 [23]	USA	NCDB	2010–2014	373,334	61	Effect on OS	Compared to <4 weeks, surgery performed >8 weeks was associated with worse 5-year OS
**Surgery following neoadjuvant therapy (TTS-n)**							
Sanford RA 2015 [22]	USA	Retrospective review of prospectively collected data	1995–2007	1101	NA	Effect of TTS (≤4, 4–6, >6 weeks) after NAC on OS	TTS after NAC up to 8 w has no effect on OS, RFS or LRFS. Worse OS if >8 weeks
Al-Masri M 2021 [26]	Jordan	Retrospective review	2006–2014	468	NA (65.4% ≤50-Y)	Effect of TTS (<4, 4–8, >8 weeks) after NAC on OS and DFS	TTS after NAC up to 8 w has no effect on OS or DFS. Worse OS if >8 w
Sutton TL 2020 [27]	USA	Retrospective review of prospectively collected data	2011–2017	463	NA	Impact of TTS (≤4, 4–6, >6 weeks) after NAC on RFS, OS, DSS and RCB scores	TTS > 6 w associated with worse RFS and DSS, and a higher RCB score
Cullinane C 2021 [32]	Ireland	Systematic review and meta-analysis. Five studies met inclusion criteria		8794	44–56	Effect of TTS (<4, 4–8, >8 weeks) post-NAC on OS, DFS and pCR	TTS < 8 weeks associated with better OS and DFS compared to TTS > 8 weeks. Equivalent pCR between TTS < 4 w and 4–8 weeks

DFS: Disease-free survival; DSS: disease-specific survival; HR: hazard ratio; NAC: neoadjuvant chemotherapy; NCDB: National Cancer Database; OS: overall survival; RFS: relapse-free survival; pCR: pathological complete response; RCB: residual cancer burden; SEER: Surveillance Epidemiology and End Results; TNBC: triple-negative breast cancer; TTS: time to surgery; Yr: year.

**Table 2:** Time to chemotherapy (TTC).

Author/Year [References]	Country	Design	Study Period	No. of Patients	Age (Median/Mean)	End Point(s)	Findings
Gagliato Dde M, 2014 [33]	USA	Retrospective review	1997–2011	6827	50	Association between TTC (≤30, 31–60, and ≥61 d) after surgery and 5-year OS, RFS and DRFS	Stage I: no difference. Stage II: worse DRFS for ≥31 d. Stage III: worse OS, RFS and DRFS for TTC ≥ 61 d. TNBC and HER2 tumors had worse OS for TTC ≥ 61 d
Kupstas AR, 2019 [34]	USA	Retrospective analysis of prospectively collected NCDP data	2010–2014	172,043	55	Impact of surgery type on TTC, and impact of TTC (< or > 120 d) from diagnosis on OS	Chemotherapy initiation >120 d after diagnosis was associated with poorer OS. Time from diagnosis to surgery had the greatest impact on time from diagnosis to chemotherapy. Reconstructive surgeries resulted in the greatest delays
Morante Z 2021 [35]	Peru	Retrospective review	2000–2014	687	48	Impact of TTC (≤30, 31–60, 61–90 and ≥91 d) after surgery on OS and DRFS for TNBC	TTC ≥ 30 d was associated with poorer OS and DRFS
Raphael MJ, 2016 [36]	Canada	Systematic review and meta-analysis. Fourteen studies met inclusion criteria	NA	NA	47–51	Relationship between TTC after surgery and OS	A 4-week increase in TTC was associated with significant increase in risk of death
Heeg E, 2020 [37]	Netherlands	Retrospective analysis of prospectively collected NCR data	2009–2014	3016		Impact of TTC > 30 d after surgery on 10-year OS	TTC > 30 d associated with worse OS in BCS patients, but not in mastectomy
Pomponio MK, 2019 [38]	USA	Retrospective analysis of prospectively collected data	2009–2018	724	55	Impact of TTC (≤31, 32–42, 43–56 and >56 d) from surgery on DFS and OS in TNBC	Mastectomy with reconstruction associated with delayed TTC. No difference in DFS or OS for TTC > 56 d vs. ≤31 d
Hershman DL, 2006 [39]	USA	Retrospective analysis of prospectively collected SEER data	1992–1999	5003	NA (all patient ≥ 65)	Factors associated with longer TTC, and impact of TTC (within 1 mo, 1–2 mo, 2–3 mo and >3 mo) on OS and CSS	Delays associated with advanced age, early stage, rural location, unmarried, mastectomy, HR-positive and no radiotherapy. Worse OS and CSS for TTC >3 mo, no difference for TTC < 1–3 mo

CSS: Cancer-specific survival; d: days; DFS: disease-free survival; DRFS: distant recurrence-free survival; HR: hazard ratio; mo: months; OS: overall survival; RFS: relapse-free survival; TNBC: triple-negative breast cancer; TTC: time to chemotherapy; Yr: year.

**Table 3:** Time to radiation therapy (TTR).

Author/Year [References]	Country	Design	Study Period	No. of Patients	Age (Median/ Mean Age)	End Point(s)	Findings
Nixon AJ, 1994 [40]	USA	Retrospective review	1968–1985	653	47/49	Relationship between TTR (0–4 w, 5–8 w and 9–12 w) and recurrence risk in stage I-II, pN0, BCS patients with no intervening chemotherapy	No difference in 5-year failures up to TTR of 8 weeks
Raphael MJ, 2020 [43]	Canada	Retrospective review	2001–2002	1028	52.2/64.5	Effect of TTR on EFS for stage I-II breast ca. post-BCS and RT	Worse EFS for TTR ≥ 12 w from surgery if no CT, and for TTR ≥ 6 w from last CT if there is intervening CT
Cao L, 2021 [44]	China	Retrospective review	2009–2015	989	52	Effect of TTR (<4, 4–8, 8–12 and >12 w) after adjuvant CT on survival outcomes for stages I-III breast ca.	TTR > 12 w post-CT resulted in inferior OS and BCSS, especially in HR-positive tumors, positive LNs and those receiving mastectomy
Gupta S, 2016 [45]	Canada	Systematic review and metanalysis	1975–2015	For LR: 13,291 from 10 publications. For OS: 2207 from 4 publications	NA	Effect of TTR on LR and OS post-BCS and RT	Increase risk of LR for each month of delay
Hébert-Croteau N, 2004 [46]	Canada	Retrospective review	1988–1994	1062	NA (70.5% < 65 years)	Effect of TTR on OS, LRFS and DDFS for stage I-II node-negative breast ca. post-BCS and RT	TTR > 12 w after surgery compromised local control with no effect on survival

BCS: Breast-conserving surgery; BCSS: breast cancer-specific survival CT: chemotherapy; DDFS: distant disease-free survival; EFS: event-free survival; HR: hormone receptors; LR: local recurrence; LRFS: local relapse-free survival; OS: overall survival; RT: radiotherapy.

## Data Availability

Not applicable.
